# Chronic Inflammation Increases the Sensitivity of Mouse Treg for TNFR2 Costimulation

**DOI:** 10.3389/fimmu.2017.01471

**Published:** 2017-11-07

**Authors:** Tobias Schmid, Lena Falter, Sabine Weber, Nils Müller, Konstantin Molitor, David Zeller, Dorothea Weber-Steffens, Thomas Hehlgans, Harald Wajant, Sven Mostböck, Daniela N. Männel

**Affiliations:** ^1^Institute of Immunology, University of Regensburg, Regensburg, Germany; ^2^Institute of Immunology, Regensburg Center for Interventional Immunology (RCI), University Medical Center, Regensburg, Germany; ^3^Division of Molecular Internal Medicine, Department of Medicine II, University Hospital Würzburg, Würzburg, Germany

**Keywords:** inflammation, immune regulation, costimulation, MDSC, TNFR2, regulatory T cell

## Abstract

TNF receptor type 2 (TNFR2) has gained attention as a costimulatory receptor for T cells and as critical factor for the development of regulatory T cells (Treg) and myeloid suppressor cells. Using the TNFR2-specific agonist TNCscTNF80, direct effects of TNFR2 activation on myeloid cells and T cells were investigated in mice. *In vitro*, TNCscTNF80 induced T cell proliferation in a costimulatory fashion, and also supported *in vitro* expansion of Treg cells. In addition, activation of TNFR2 retarded differentiation of bone marrow-derived immature myeloid cells in culture and reduced their suppressor function. *In vivo* application of TNCscTNF80-induced mild myelopoiesis in naïve mice without affecting the immune cell composition. Already a single application expanded Treg cells and improved suppression of CD4 T cells in mice with chronic inflammation. By contrast, multiple applications of the TNFR2 agonist were required to expand Treg cells in naïve mice. Improved suppression of T cell proliferation depended on expression of TNFR2 by T cells in mice repeatedly treated with TNCscTNF80, without a major contribution of TNFR2 on myeloid cells. Thus, TNFR2 activation on T cells in naïve mice can lead to immune suppression *in vivo*. These findings support the important role of TNFR2 for Treg cells in immune regulation.

## Introduction

TNF is a key inflammatory cytokine regulating the immune system. It induces inflammation and tissue injury via the activation of TNF receptor type 1 (TNFR1). Currently, TNF blockade is used as anti-inflammatory intervention in patients with chronic inflammatory diseases such as rheumatoid arthritis or inflammatory bowel diseases (1–3). However, there is also evidence for adverse side effects from experimental and clinical studies (4–7). The interaction of TNF with its two functionally different receptors TNFR1 and TNF receptor type 2 (TNFR2) partly explains the complexity of TNF effects. Selective inhibition of soluble TNF or of TNFR1 has been suggested to avoid detrimental TNFR1 activation but to preserve the interaction of endogenous membrane TNF with TNFR2 (8). Activation of TNFR2 has gained attention, in particular, in conferring immune suppression (9) and, recently, by inducing regulatory T (Treg) cells (10–13). The T cell costimulatory effect of several TNF family members, including TNFR2, seems to be important for the promotion of the development of Treg cells (14). Also, expansion of suppressive Treg cells *in vitro* was improved by activation of TNFR2 (15, 16). Thus, TNFR2 proved to be critically involved in generation and function of regulatory T (Treg) cells, offering the opportunity for a more specific immune regulatory treatment of autoimmune diseases (13, 17, 18).

The role of TNFR2 in immune suppression conferred by myeloid-derived suppressor cells (MDSC), a not so well characterized immature subpopulation of myeloid cells, is less clear. Generation of functional MDSC seems to depend on TNFR2 signaling by arresting their differentiation to mature macrophages (19, 20). In addition, activation of TNFR2 is also required for the optimal suppressive function of MDSC (21, 22).

We and others have previously shown that TNFR2 signaling impacts both on T cell and myeloid cell populations. So far, however, no specific activation of the TNFR2 was applied, but indirect models of TNFR2-deficiency were used. Here, we present a study of effects induced by a TNFR2-specific agonist on the cellular level. The contribution of TNFR2 activation on T cells, Treg cells, and MDSC was analyzed *in vitro* as well as *in vivo* in naïve mice and in mice with chronic inflammation. This comparative study of healthy and diseased animals with focus on multiple immune cell populations aims at a better assessment of the TNFR2 agonist as a possible therapeutic agent. While TNFR2 signaling is crucial for induction of suppressive Treg cells (10–13), we show here that, by contrast, activation of TNFR2 on myeloid cells interfered with the maturation of MDSC and reduced their suppressive capacity. However, expression of TNFR2 on T cells was critical for the dominating immune suppressive effect of TNFR2 agonist in chronically inflamed mice. Thus, the level of inflammation and therefore the targeted pathology seem to be critical parameters for the therapeutic use of the TNFR2 agonist.

## Materials and Methods

### Mice

C57BL/6 mice were purchased from Janvier (LeGenest, France). TNFR2-deficient mice (C57BL/6-Tnfrsf1btm1Mwm) (23) were purchased from The Jackson Laboratory (Bar Harbor, ME, USA). C57BL/6N Ly5.1 (CD45.1) (24) mice were kindly provided by Petra Hoffmann, University of Regensburg. Mice carrying the conditional TNFR2^flox/flox^ allele (TNFR2^fl/fl^) were generated by breeding Tnfrsf1b/tm1a(EUCOMM)Wtsi mice to FLPe delete mice (25). Location and orientation of both loxP sites and deletion of the beta-galactosidase reporter gene and the neomycin resistance cassette were verified by cloning of the corresponding PCR products and subsequent sequence analysis. For genotyping the following primers were used: 5′ TGTGAGTGCAAGGACACACGGTGC 3′ and 5′ GGCCAGGAAGTGGGTTACTTTAGGGC 3′. Cell-specific ablation of TNFR2 on T cells (CD4cre/TNFR2^fl/fl^) was achieved by breeding TNFR2^fl/fl^ mice to CD4-Cre mice (26). CD4cre/TNFR2^fl/fl^ lack the expression of TNFR2 on T cells while the expression on myeloid cells is not changed. To generate macrophage- and neutrophil-specific TNFR2-deficient mice (LysMcre/TNFR2^fl/fl^), TNFR2^fl/fl^ mice were crossed with LysM-Cre mice (27). Fewer myeloid cells express TNFR2 in these mice and the expression is mainly seen on immature myeloid cells of the MO-MDSC subtype. Mice were bred and housed in an animal facility with barrier conditions at the University of Regensburg. This study was carried out in accordance with institutional guidelines. The protocol was approved by the district government of Lower Franconia, Würzburg (Az: 54-2532.1-27/10, AZ: 54-2532.1-37/13).

### TNFR2 Agonist

Generation of tenascin-trimerized single-chain mouse TNF receptor p80 (TNFR2)-specific TNF (TNCscTNF80) as a TNFR2-specific agonist has been described recently as STAR2 (13). The TNCscTNF80 expression cassette was subcloned into pT2/SV-Neo and transfected into HEK293 cells together with the Sleeping Beauty Transposon plasmid pCMV(CAT)T7-SB100 [Addgene, Cambridge, MA, USA (28)] to produce TNCscTNF80 from HEK293 transfectants. TNCscTNF80 contains a Flag epitope and was purified from cell supernatants by affinity chromatography on anti-FlagM2 Agarose and eluted with Flag-peptide (Sigma, Deisenhofen, Germany). After dialysis (Spectra/Por, Serva, Heidelberg, Germany), the protein concentration was determined by scanning (Typhoon 9200, GE Health Care, Solingen, Germany) a SyproRed (Invitrogen, Carlsbad, CA, USA)-stained polyacrylamide gel (10% SDS-PAGE) and comparing the intensity of the TNCscTNF80 band with that of a BSA protein standard (Invitrogen, Life Technologies, Darmstadt, Germany) using the Image Quant TL 7.0 Analysis software (GE Health Care). Biological activity and specificity was routinely tested in a T cell proliferation costimulator test: carboxyfluorescein succinimidyl ester (CFSE, eBioscience, Frankfurt, Germany)-labeled spleen cells (2 × 10^6^/ml) were cultured with anti-CD3 (0.1 µg/ml) with or without TNCscTNF80 (50 and 5 ng/ml) for 72 h. Proliferation of CD4 and CD8 T cells was quantified by FACS analysis. Lipopolysaccharide contamination was excluded in control experiments with heat-inactivated TNCscTNF80. TNCscTNF80 exclusively and specifically binds to and activates TNFR2 but not TNFR1 (13).

### Cells

Cell separation out of cell suspensions was performed with magnetic beads following the instructions of the manufacturer (Miltenyi Biotec GmbH, Bergisch Gladbach). Bone marrow-derived myeloid cells were generated from bone marrow as described (29). For evaluation of NO production capacity, these cells were stimulated with LPS (*E. coli* O127:B8, 0.1 µg/ml, Sigma) and IFNγ (120–240 IU/ml, PeproTech GmbH, Hamburg) for 48 h.

### Flow Cytometry

Single cell suspensions were prepared from spleens, and pooled lymph nodes and red blood cells were lysed, or cells were harvested from cell culture. Unspecific antibody binding was blocked by anti-FcRII/III-antibody (BD Biosciences, Heidelberg, Germany) and cells stained with fluorochrome-labeled antibodies. Antibodies for flow cytometric analyses were purchased from either eBiosciences (Frankfurt, Germany) or BD Bioscience. Fluorescence was measured on a BD LSR-II cytometer and analyzed using FACSDiva software (BD Biosciences). Living single cells were gated based on forward/sideward scatter properties.

### T Cell Proliferation

Single cell suspensions from spleens were prepared, and red blood cells lysed. For proliferation assays, splenocytes were labeled with 1 µM CFSE (Invitrogen). CFSE-labeled splenocytes (2 × 10^5^) or purified T cells (5 × 10^4^) were activated with 0.1 µg/ml anti-CD3ε antibody (clone 145.2C11, purified from hybridoma supernatant) with or without additional stimulation as indicated for 72 h. In an experiment to test the requirements for CD28, T cells were purified from splenocytes before CFSE-labeling. CFSE-labeled T cells were then stimulated with 0.5 µg/ml anti-CD3ε antibody, with or without 2.5 µg/ml anti-CD28 (clone 37.51), with or without blocking anti-CD80 (clone 16-10A1, 10 µg/ml) + anti-CD86 (clone GL-1, 10 µg/ml) antibodies. Cell proliferation was analyzed after 72 h by assaying CFSE dilution by flow cytometry.

### Treg Cell Expansion

Treg cells with a purity of more than 98% CD4^+^CD25^high^CD62L^+^ from wild-type as well as TNFR2^−/−^ mice were cultured in the presence of anti-CD3ε and anti-CD28 antibodies (MACSiBead particles, Miltenyi Biotec) and recombinant human IL-2 (Proleukin S, Novartis Pharma, Basel, Switzerland) with or without TNCscTNF80 according to the instructions of the manufacturer of the Treg cell expansion kit (mouse, Miltenyi Biotec) for 7 days.

### T Cell Suppression

Carboxyfluorescein succinimidyl ester-labeled effector spleen cells (0.5–1.5 × 10^5^) were cultured with or without Treg cells for 72 h as described (10, 30). Labeled effector cells from TNFR2-deficient mice were used to avoid interferences by activation of the TNFR2 on effector cells in the cultures. Duplicate or triplicate cultures were stimulated with soluble anti-CD3ε antibodies (0.5 µg/ml, BD Bioscience) for 72 h. Interleukin 10 (IL-10) was quantified using the Duo Set ELISA (R&D Systems, Minneapolis, MN, USA) according to the manufacturer’s instructions. To determine the suppressive activity of myeloid suppressor cells, CFSE-labeled spleen cells (2 × 10^5^) were stimulated with anti-CD3ε (0.25 µg/ml, BD Bioscience) and anti-CD28 (0.125 µg/ml, BD Bioscience) and cultured with or without different numbers of bone marrow-derived myeloid cells. Proliferation of CD4 or CD8 T cells was determined by flow cytometry. Nitrite concentrations in the supernatants were determined using Griess reagent measuring the optical density at 540 nm.

### *In Vivo* Analysis of T Cell Activation

Naïve TNFR2^fl/fl^, CD4cre/TNFR2^fl/fl^, LysMcre/TNFR2^fl/fl^, and TNFR2^−/−^ mice were injected six times (ip) with either TNCscTNF80 (75 µg/mouse) or PBS every other day. Two days after treatment cessation, between 1.5 × 10^6^ and 1 × 10^7^ CFSE-labeled T cells from untreated wild-type mice were adoptively transferred and *in vivo* activated with anti-CD3ε antibody (10 µg/mouse, purified from hybridoma supernatant of clone 145.2C11, iv) the next day. Three days later, splenocytes and the axial, brachial, and inguinal lymph node cells were analyzed by flow cytometry for T cell proliferation.

### Model of Chronic Inflammation

To induce chronic inflammation in mice, the model described by Sade-Feldman was used (20). In brief, mice were immunized three times with *Mycobacterium tuberculosis*-BCG (231141, 50 µg, Difco Laboratories, Detroit) in incomplete Freund’s adjuvant (Sigma) every 7 days with the last injection without Freund’s adjuvant. Two days later, mice were analyzed.

### Statistical Analyses

GraphPad Prism 5 was used as statistical software for the data analyses. For comparing two groups, Student’s *t*-test was used. For comparing multiple groups, one-way ANOVA with Tukey *post hoc* test, or two-ANOVA with Bonferroni *post hoc* test were used. For analysis of experiments with several dilutions of anti-CD3-antibody, the area-under-the-curve was calculated and used for the statistical analysis. Statistical significant results (*p* < 0.05) are indicated with asterisks (*) in the figures.

## Results

### Effect of TNFR2 Activation on T Cells

**Figure 1:**
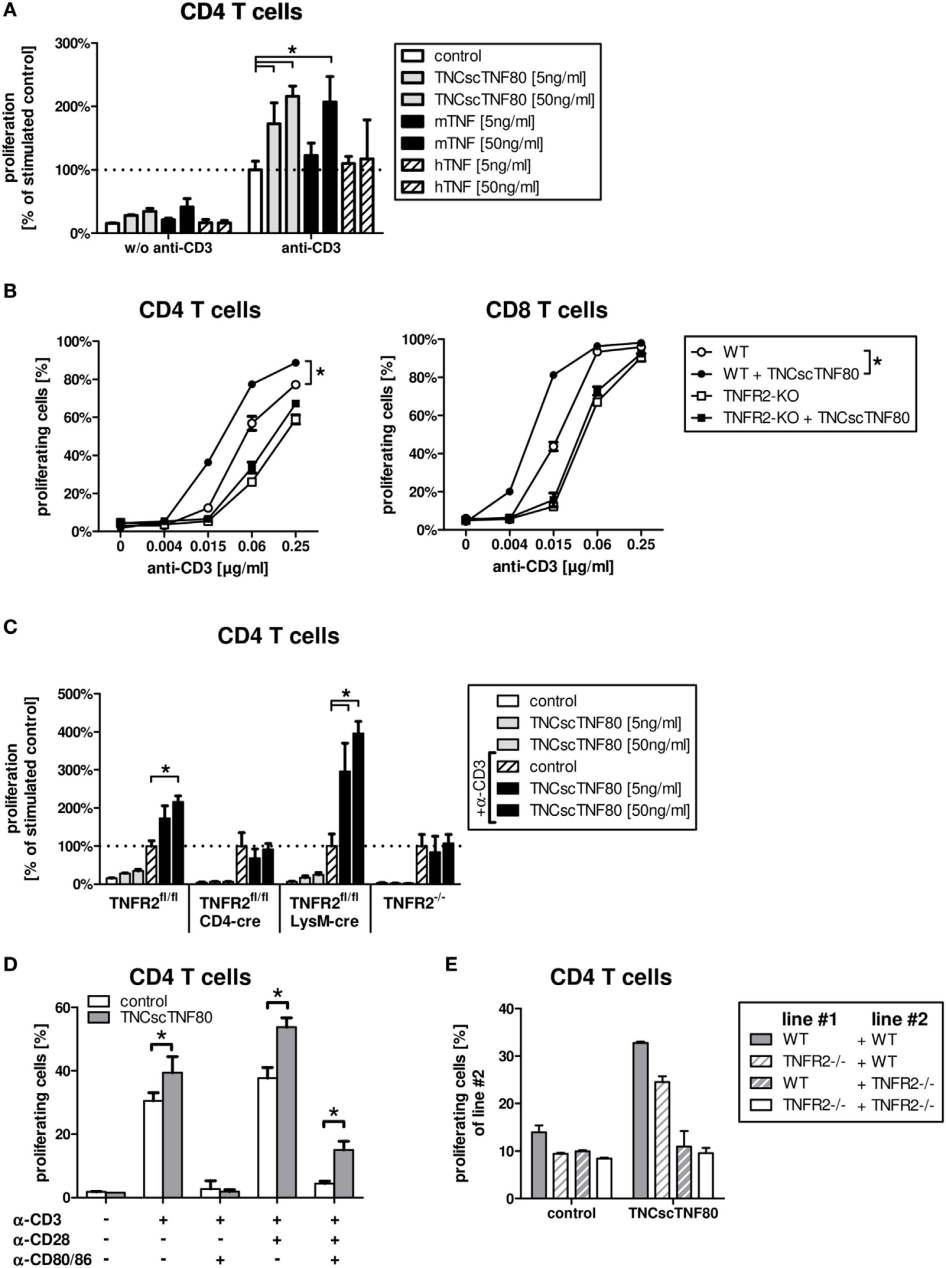
TNCscTNF80-increased proliferation *in vitro*. The relative proliferation of stimulated CD4 T cells from WT mice without or with 5 or 50 ng/ml of either TNCscTNF80, mouse TNF, or human TNF is shown. Data are relative to anti-CD3ε-activated T cells without any TNF variant. **(A)** The proliferation of CD4 (left) or CD8 (right) T cells from either WT (round symbols) or TNFR2-deficient (square symbols) mice is shown in the presence (filled symbols) or absence (empty symbols) of TNCscTNF80 (10 ng/ml) and increasing concentrations of stimulating anti-CD3ε antibodies. Data shown are mean + SD of culture replicates from one representative experiment of five. For statistical analysis, the area-under-the-curve was calculated for all five experiments, and the combined data were analyzed. **(B)** The relative proliferation of stimulated CD4 T cells from different mouse lines without or with 5 or 50 ng/ml of TNCscTNF80 is shown. For each mouse line, proliferation was calculated relative to the respective control of anti-CD3ε-activated T cells without TNCscTNF80 **(C)**. Purified CD4 T cells from WT mice were stimulated with various combinations of anti-CD3ε (0.5 µg/ml), anti-CD28 (2.5 µg/ml) antibody, and blocking anti-CD80/CD86 antibodies (10 µg/ml). The proliferation of CD4 T cells cultured with or without TNCscTNF80 (10 ng/ml) is shown **(D)**. Splenocytes from WT and TNFR2-deficient mice were split in two parts, one labeled with carboxyfluorescein succinimidyl ester (CFSE) and one unlabeled. Unlabeled splenocytes (line #1) were combined with CFSE-labeled splenocytes (line #2) at a ration of 1:1 and stimulated with anti-CD3ε antibody. The proliferation of CFSE-labeled CD4 T cells (line#2) in the presence or absence of TNCscTNF80 (10 ng/ml) is shown **(E)**. For panels **(A,C–E)**, mean + SD of culture replicates from one out of two independent experiments is given.

Treatment with a recombinant agonistic fusion protein (TNCscTNF80) with selective activity for mouse TNFR2 induced expansion of Treg cells in mice as reported recently (13). To analyze the effect in more detail on a cellular and molecular level, we tested TNCscTNF80 for costimulation of T cell activation. The costimulatory effect of TNF can be measured as facilitated induction of T cell proliferation and has been described to be TNFR2-specific (31, 32). Mouse spleen cells were cultured with limiting concentrations of anti-CD3ε agonistic antibody (to activate the TCR) in the presence or absence of recombinant human TNF, mouse TNF, or TNCscTNF80, and the proliferation of CD4 and CD8 T cells was analyzed. Figure 1 shows data of CD4 T cells; CD8 T cells responded in a similar way (data not shown). Consistent with the concept of TNFR2 as a costimulatory receptor, TNCscTNF80 induced proliferation of T cells (Figure 1A) only in combination with parallel activation of the TCR. Such requirement of TCR activation for the effect of TNCscTNF80 on T cells has already been described recently (13). Costimulation by TNCscTNF80 was superior over mouse TNF at low protein concentrations. Human TNF, known not to activate the mouse TNFR2 (31, 33), did not affect T cell proliferation. TNCscTNF80 improved the proliferation of CD4 as well as CD8 T cells from wild-type mice in a dose-dependent fashion (Figure 1B). CD8 T cells reacted at about four times lower anti-CD3 concentrations than CD4 T cells to the TNFR2 costimulation. TNCscTNF80 had no costimulatory effect on T cells from TNFR2-deficient mice confirming the TNFR2-specificity of the agonistic agent (Figure 1C). TNCscTNF80 in the presence of 0.5 µg/ml anti-CD3ε also strongly enhanced the IFNγ release in spleen cell cultures from 28.8 pg/ml in controls without TNCscTNF80 to 1,165.2 pg/ml in cultures with TNCscTNF80 (10 ng/ml).

**Figure 2:**
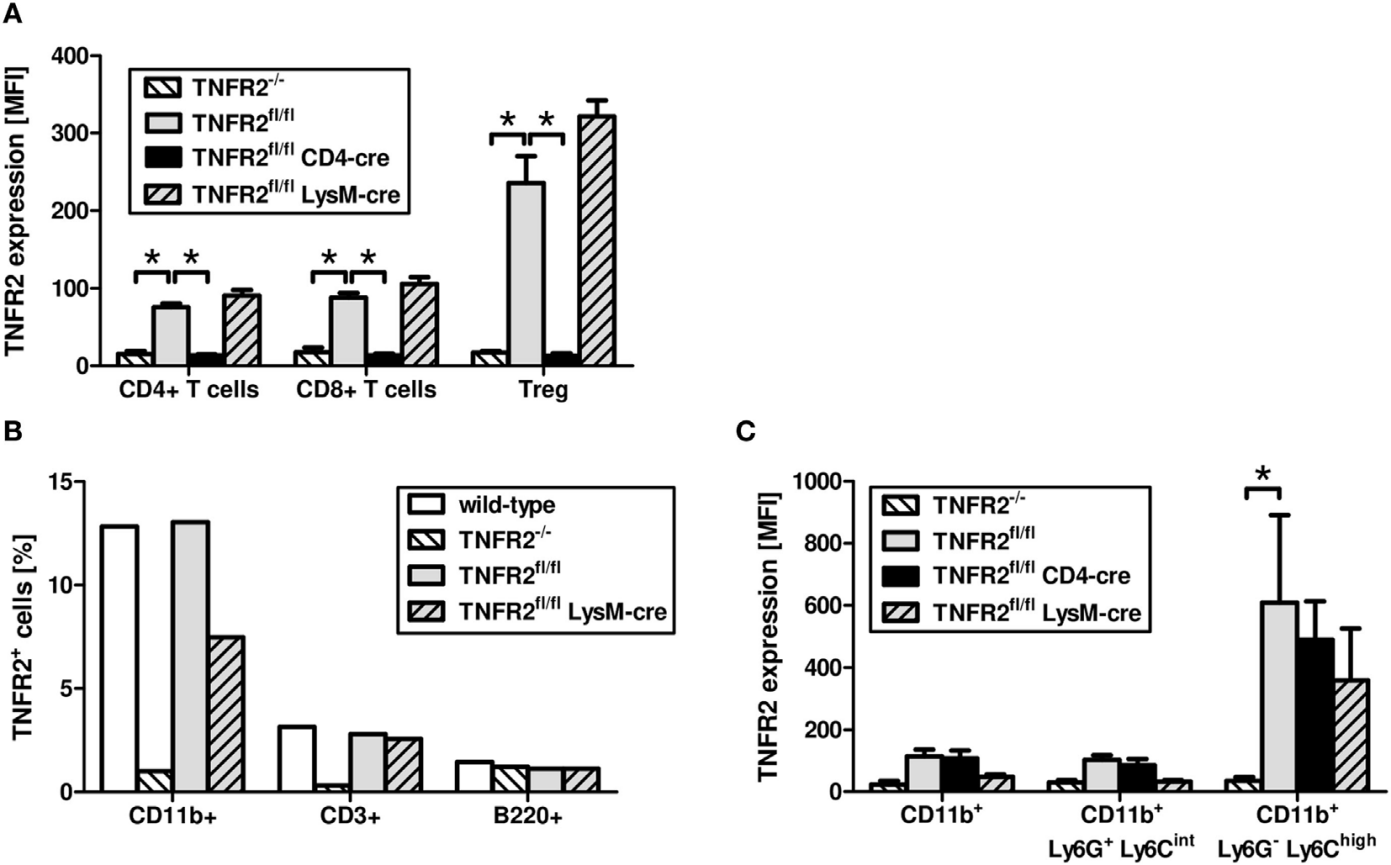
Expression of TNFR2 on cells from genetically modified mouse lines. Splenocytes of naïve male mice of each mouse genotype were analyzed by flow cytometry for the expression levels [mean fluorescent intensity (MFI)] of TNFR2 on CD4^+^, CD8^+^, and Treg (CD4^+^Foxp3^+^) cells **(A)**. The frequency of cells expressing TNFR2 on CD11b^+^, CD3^+^, and B220^+^ cells was analyzed by flow cytometry **(B)**. Bone marrow cells of naïve male mice of each mouse genotype were analyzed by flow cytometry for the MFI of TNFR2 expression on two subtypes of CD11b^+^ myeloid cells, PMN-MDSC (Ly6G^+^Ly6C^int^), and MO-MDSC (Ly6G^−^Ly6C^high^) **(C)**. Results derived from three individual mice per group are expressed as mean values + SD. The data are representative of one out of three experiments.

Similar to T cells from TNFR2-deficient mice, T cells from CD4cre/TNFR2^fl/fl^ mice, lacking TNFR2 on all T cells (Figure 2A), did not react to TNCscTNF80 costimulation (Figure 1C). By contrast, T cells from LysMcre/TNFR2^fl/fl^ mice—with reduced TNFR2 expression on myeloid cells and in particular on immature myeloid cells of the MO-MDSC subtype (Figures 2B,C)—showed a proliferative pattern similar to wild-type T cells upon TNCscTNF80 application.

The costimulatory effect of TNCscTNF80 for T cell proliferation was prevented by blocking the activation of CD28 by using anti-CD80 antibodies, demonstrating that TNCscTNF80 was not able to compensate for lack of CD28 activation (Figure 1D).

The requirement for direct activation of TNFR2 on T cells for costimulation by TNCscTNF80 was validated further using mixed cultures of splenocytes from wild-type and TNFR2-deficient mice. The proliferation of TNFR2-deficient T cells was not enhanced by TNCscTNF80 even in cultures also containing T cells from wild-type mice (Figure 1E).

### Effect of TNFR2 Activation on Treg Cell Function

**Figure 3:**
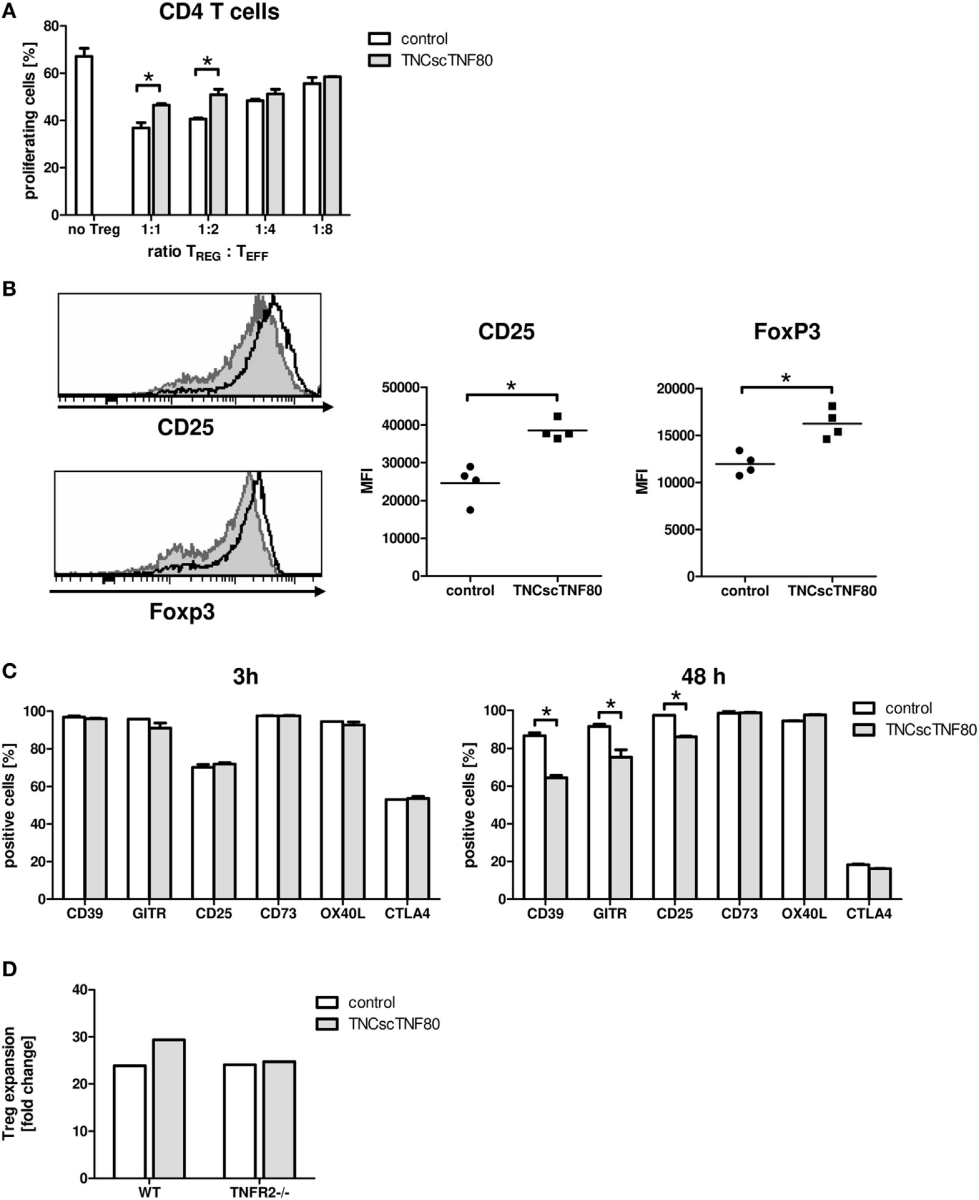
Effects of TNCscTNF80 on Treg cells. Treg cells from wild-type (CD45.1) mice were cultured with anti-CD3 activated TNFR2-deficient splenocytes (CD45.2) with or without TNCscTNF80 (10 ng/ml) at the indicated ratios. The percentage of proliferating CD4 T effector cells (CD45.2) was determined by flow cytometry after 72 h. Data are given as mean of culture replicates + SD from one out of two independent experiments **(A)**. Treg cells from four CD45.2 mice were separately cultured with activated CD45.1 splenocytes with or without TNCscTNF80 (10 ng/ml). After 72 h, the expression profile (left) and the mean of the fluorescent intensity (MFI, right) for CD25 and Foxp3 of Treg cells (CD45.2) from these cultures were analyzed. The left panels show the marker expression profile of CD45.2 Treg cells in one representative culture without (gray histograms) and one culture with (black line histograms) TNCscTNF80. In the right panels, each symbol represents one mouse. Data from one out of four independent experiments are shown **(B)**. Activated total splenocytes were cultured for 3 or 48 h with (gray bars) or without (white bars) TNCscTNF80 (10 ng/ml), and the expression of the surface markers CD39, GITR, CD25, CD73, OX40L, and CTLA-4 were determined on Treg cells (CD4^+^Foxp3^+^) by flow cytometry. Data from one experiment are given as mean of culture replicates + SD **(C)**. Purified CD4^+^CD25^high^CD62L^+^ Treg cells from wild-type as well as TNFR2-deficient (TNFR2^−/−^) mice were cultured in the presence of antibodies to anti-CD3ε, anti-CD28, and recombinant human IL-2 with or without TNCscTNF80. After 7 days, the cell yield of Treg (CD4^+^Foxp3^+^) cells from wild-type and TNFR2-deficient mice was determined, and the expansion calculated. Representative data of one experiment out of two with similar results are shown **(D)**.

To test the influence of the TNFR2 agonist on the suppressive function of Treg cells, TNCscTNF80 was added to Treg cell-containing cultures of proliferating T cells. Cells from TNFR2-deficient mice were used as CFSE-labeled effector T cells to avoid any costimulatory effect by the activation of the TNFR2 on the effector T cells. Added to such suppression cultures, TNCscTNF80 diminished the Treg-induced suppression of CD4 T cells (Figure 3A). In this experimental setup, CD8 T cells were not suppressed by Treg cells. Hence, a possible reduction of CD8 T cell suppression was not assessed.

To further study the impact of TNCscTNF80 on Treg cells, total splenocytes were cultured in the presence of agonistic anti-CD3ε antibody. Interestingly, phenotypic analysis demonstrated that TNCscTNF80 increased the expression levels of CD25 and Foxp3 of Treg cells after 72 h in these cultures (Figure 3B). However, TNCscTNF80 downregulated the percentages of Treg cells positive for CD39, GITR, or CD25 within 48 h, while CD73, OX40L, and CTLA-4 were not affected. No effects were observed at 3 h of culture (Figure 3C). Furthermore, we did not observe an impact on TNFR2 levels of Treg in such cultures (data not shown). The TNFR2 agonist had marginal effects on the IL-2-induced activation of STAT5 and did not affect pZAP70 in Treg cells during T cell activation (data not shown). This is in full agreement with results of Kim et al. suggesting discrete effects of TNFR2 on the signaling pathways of T cells (2, 34, 35).

Since the presence of TNCscTNF80 seemed to improve viability and expansion of Treg cells, highly purified Treg cells were stimulated and cultured in the presence of IL-2 with or without TNFR2 agonist. As CD8 T cells would overgrow costimulated T cell cultures, single contaminating CD8 T cells were avoided by very careful sorting of purified Treg cells. Treg cells with a purity of more than 98% of CD4^+^CD25^high^CD62L^+^ cells from wild-type as well as TNFR2^−/−^ mice were cultured in the presence of anti-CD3ε and anti-CD28 antibodies and recombinant human IL-2 with or without TNCscTNF80 for 7 days. By the end of the expansion period, Treg cells had expanded 23.9-fold without TNCscTNF80. This expansion was increased to 29.4-fold by the presence of the TNFR2 agonist (Figure 3D). As expected, TNFR2-deficient Treg cells did not profit from the enhancing effect of TNCscTNF80 in the same experiment (yield 24.1 and 24.7-fold, respectively; similar effects were observed in a second experiment). The expanded cells of both groups consisted of at least 97% Treg cells and expressed similar levels of various Treg signature markers, e.g., CD25, FoxP3, and GITR (data not shown).

### Effect of TNFR2 Activation on Myeloid Cells

**Figure 4:**
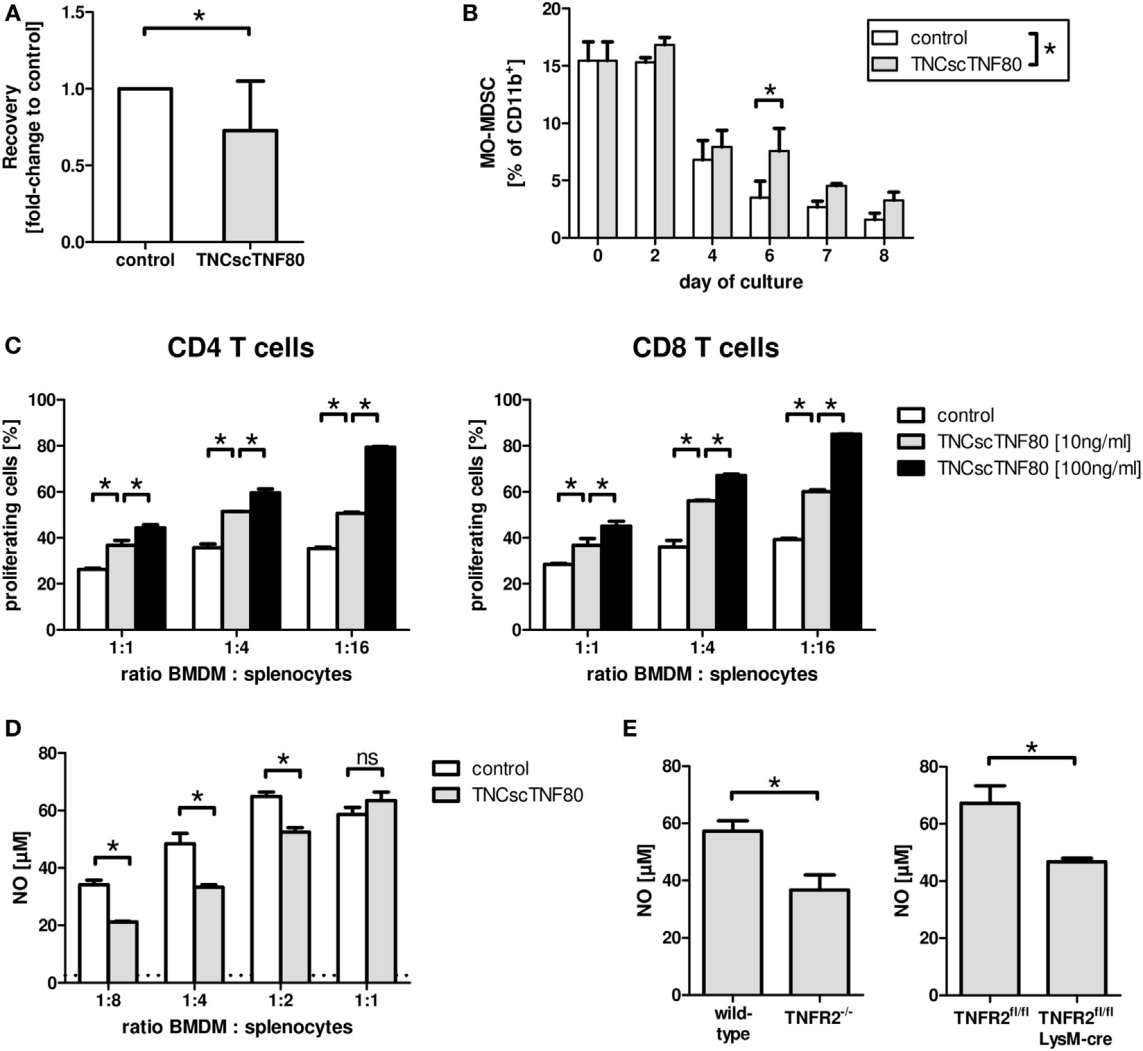
Effects of TNCscTNF80 on myeloid cells. The cell yield of bone marrow-derived myeloid cells was determined after 7 days of culture in the presence of GM-CSF with or without TNCscTNF80 (10 ng/ml). Data of 10 experiments are shown as mean + SD of the normalized values. **(A)** The percentage of immature MO-MDSC (Ly6G^−^Ly6C^high^) in cultures of bone marrow-derived myeloid cells with or without TNCscTNF80 (10 ng/ml) at the indicated time points is shown. Data shown as mean + SD of four mice from one experiment **(B)**. Suppressive activity of graded numbers of bone marrow-derived myeloid cells (BMDM) generated over 6 days in the presence or absence of TNCscTNF80 (10 and 100 ng/ml) was measured. Proliferation of CD4 (left) and CD8 T (right) cells was determined by cytometry **(C)**. The concentration of generated nitrite in supernatants of these cultures (containing 100 ng/ml of TNCscTNF80) was determined. The horizontal dotted line indicates the background NO levels of splenocytes cultured without additional BMDM. Data from one experiment are shown **(D)**. Bone marrow-derived myeloid cells from the indicated mouse lines were generated over 8 days, and the capacity to produce nitrite following stimulation with LPS and IFNγ was determined. Data shown as mean + SD of four mice from one experiment **(E)**.

TNFR2 also plays a role during the differentiation of myeloid precursor cells from the bone marrow to mature myeloid cells as has been shown previously (21). Lack of TNFR2 retarded differentiation of bone marrow cells from TNFR2-deficient mice and led to reduced suppressor activity of TNFR2-deficient immature myeloid cells for T cells. To test the influence of direct TNFR2 activation during myeloid cell differentiation, bone marrow cell cultures from wild-type mice containing GM-CSF with or without TNCscTNF80 were analyzed. Intriguingly, activation of TNFR2 by the TNFR2 agonist reduced the cellular yield and retarded the maturation of cells from such cultures in a similar way as seen with cells of TNFR2-deficient mice (Figures 4A,B). TNCscTNF80 also reduced their T cell suppressive activity (Figure 4C) and their capacity to produce nitrite (Figure 4D).

Furthermore, reduced nitrite production capacity following activation by LPS and IFNγ was found for bone marrow-derived myeloid cells from LysMcre/TNFR2^fl/fl^ compared with wild-type mice (Figure 4E). Thus, suppressive activity of myeloid suppressor cells seems to depend on TNFR2 expression on myeloid cells since bone marrow-derived suppressor cells from TNFR2-deficient mice have also been described to be less suppressive (21).

### Effect of TNFR2 Activation *In Vivo*

In the *in vitro* experiments described earlier, we have observed that TNFR2 activation has different effects on the functions of Treg cells and myeloid cells. To evaluate the *in vivo* impact of the TNFR2 on these cell populations on Treg cell function, Treg cells were isolated from wild-type (TNFR2^fl/fl^) and TNFR2-deficient mice as well as from CD4cre/TNFR2^fl/fl^ and LysMcre/TNFR2^fl/fl^ mice, and their suppressive activity was compared in a T cell suppression assay. Treg cells from TNFR2-deficient mice clearly suppressed the T cell proliferation to a lower degree compared with Treg cells derived from wild-type mice as previously shown (21). Surprisingly, TNFR2-deficient Treg cells from CD4cre/TNFR2^fl/fl^ mice suppressed T cell proliferation as good as wild-type Treg cells. By contrast, Treg cells from the LysMcre/TNFR2^fl/fl^ mice, with TNFR2-deficient myeloid cells, seemed to be less suppressive than wild-type Treg cells (data not shown). These experiments were not repeated since the *in vitro* suppression test might not reflect the *in vivo* situation.

**Figure 5:**
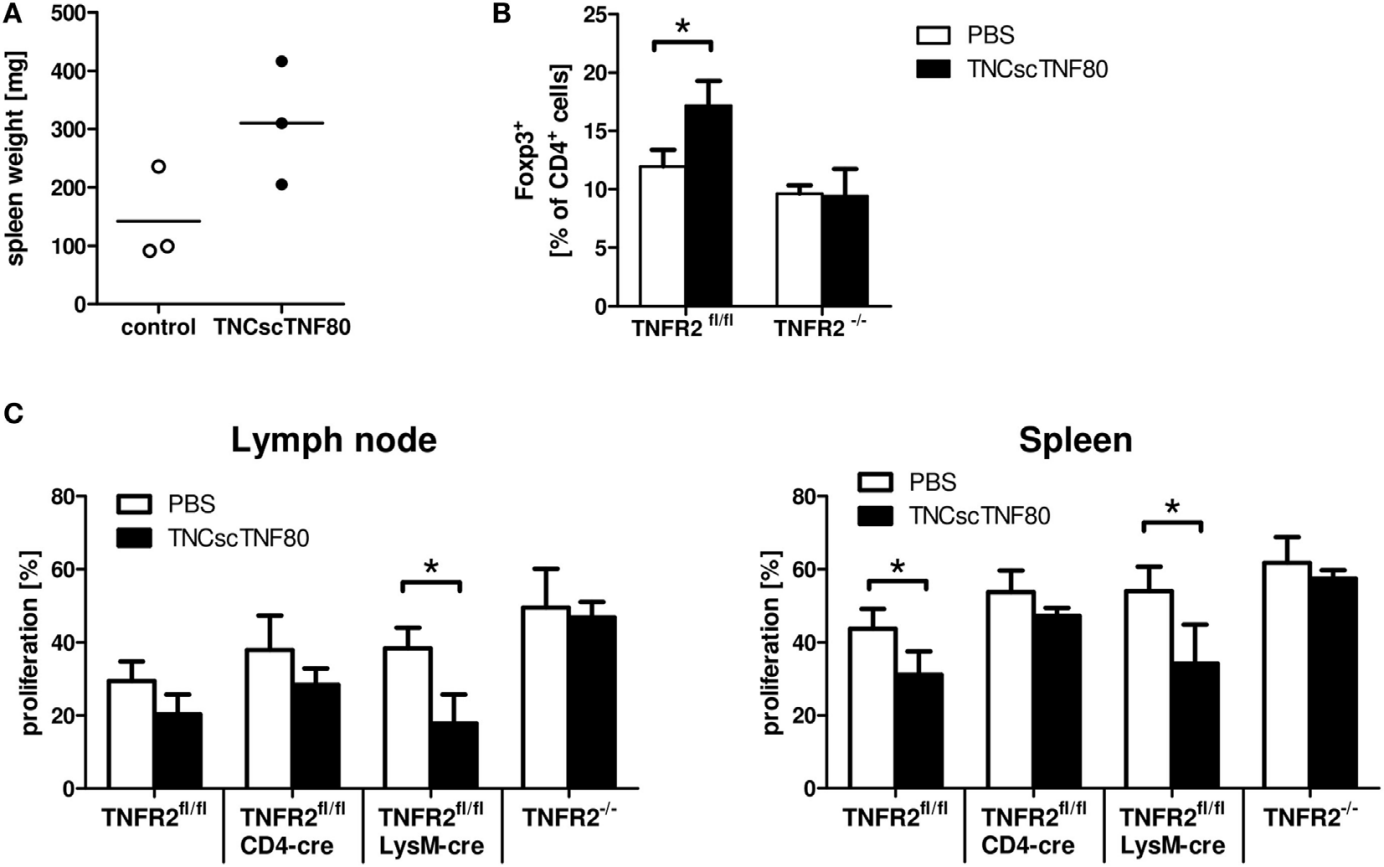
Effects of TNCscTNF80 *in vivo*. Naive mice received TNCscTNF80 (75 μg/mouse) two times (day 5 and 3). Spleen weight was determined on day 0. Data shown are from one experiment; each symbol represents a mouse **(A)**. Naïve TNFR2^fl/fl^ and TNFR2^−/−^ mice were treated six times with either TNCscTNF80 (75 μg/mouse) or PBS. Three days after treatment, the percentages of splenic Treg cells (Foxp3^+^) were analyzed by flow cytometry. Results are shown as mean + SD of three individual mice per group. Data shown are from one experiment **(B)**. Naïve TNFR2^fl/fl^, CD4cre/TNFR2^fl/fl^, LysMcre/TNFR2^fl/fl^, and TNFR2^−/−^ mice were treated six times with either TNCscTNF80 (75 μg/mouse) or PBS. Two days after treatment cessation, carboxyfluorescein succinimidyl ester (CFSE)-labeled T cells were adoptively transferred and activated. Three days later, pooled cells from axial, brachial, and inguinal lymph nodes (left) and spleen cells (right) were analyzed by flow cytometry. Percentages of proliferating cells of CFSE-positive T cells in the indicated mouse lines were determined **(C)**. Data are derived from one experiment and are shown as mean + SD of three to four individual mice per group.

To further analyze the impact of TNFR2 on leukocyte function, naïve mice were treated with TNCscTNF80. A single injection had no measurable effect on the spleen; two injections of TNFR2 agonist given within 24 h induced mild splenomegaly (Figure 5A). After two or three injections of TNCscTNF80, no change in the composition of splenocytes was observed while changes were observed in the bone marrow: mice developed first signs of increased numbers of myeloid cells after two injections, thus, indicating only a very mild peripheral inflammatory reaction to specific TNFR2 activation. A mild transient myelopoiesis was observed in the bone marrow, with an increase of CD11b^+^ myeloid cells from 42.4% in untreated mice to 62.0% in TNCscTNF80-treated mice, of CD11b^+^Ly6G^+^ immature myeloid cells from 26.2 to 43.2%, and of CD11b^+^Ly6C^high^ from 4.1 to 7.1%, respectively, after three injections of TNFR2 agonist.

When TNCscTNF80 was injected six times every other day into naïve mice, significantly enhanced spleen weight and increased myelopoiesis in the bone marrow were observed. In addition, repeated TNFR2 activation expanded Treg cells in the spleen and lymph nodes in a TNFR2-dependent manner (Figure 5B and data not shown). To test whether such mice after repeated TNCscTNF80 treatment are immune suppressed, wild-type T cells were adoptively transferred into different mouse lines that do not express TNFR2 on specific cell types. Proliferation of T cells was only reduced in wild-type (TNFR2^fl/fl^) and in LysMcre/TNFR2^fl/fl^ recipient mice. TNCscTNF80 treatment did not lead to a reduction of T cell proliferation in case of ablated systemic TNFR2 expression in TNFR2^−/−^ mice or T cell-specific TNFR2 deficiency in CD4cre/TNFR2^fl/fl^ mice (Figure 5C). These findings indicate that TNFR2 on host T cells but not host myeloid cells is required for immune suppression.

**Figure 6:**
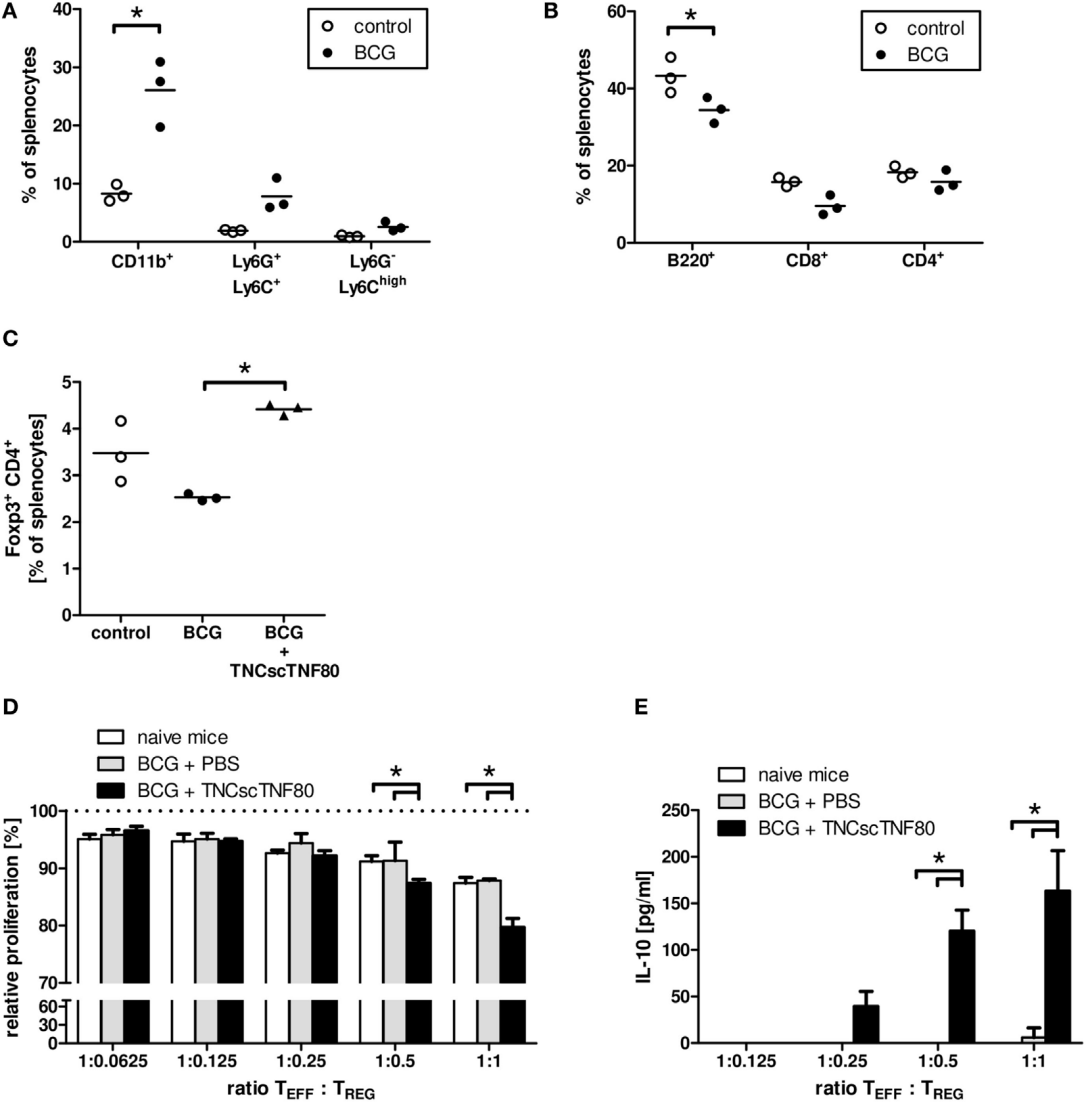
Effects of TNCscTNF80 in the BCG *in vivo* model of chronic inflammation. The composition of spleen cells from mice immunized with BCG was analyzed. The fractions of myeloid (CD11b^+^) cells and the immature myeloid cell populations PMN-MDSC (Ly6G^+^Ly6C^+^) and MO-MDSC (Ly6G^−^Ly6C^high^) **(A)**, as well as of B (B220^+^) and T (CD8^+^, CD4^+^) **(B)** were determined by flow cytometry. The fraction of Treg (CD4^+^Foxp3^+^) cells in control mice and in BCG-immunized mice treated or not with TNFscTNF80 was determined by flow cytometry **(C)**. Each symbol represents one mouse. Data are shown from one experiment out of two **(A–C)**. The relative proliferation of activated T cells in the presence of graded numbers of Treg cells from either naïve mice (open bars), BCG-treated mice (gray bars), or BCG and TNCscTNF80-treated mice (black bars) was determined. Data are shown as mean + SD of three mice from one experiment. Proliferation data are normalized to proliferation in cultures without additional Treg cells **(D)**, and concentrations of IL-10 were determined in the supernatants **(E)**.

To test the *in vivo* effects of TNFR2 stimulation in mice with ongoing inflammation, the model of chronic inflammation by Sade-Feldman was used (20). In this model, a challenge with BCG induces a strong splenomegaly in BCG-pretreated mice. The expansion of the myeloid cell fraction in bone marrow (data not shown) and spleen (Figure 6A) was paralleled by a compression of the lymphocyte compartment (Figure 6B) documenting ongoing myelopoiesis. BCG pretreatment of mice did not change the frequency of Treg cells in the CD4 T cell population compared with untreated mice (Figure 6C). Similar effects of BCG immunization were found in TNFR2-deficient mice and in mice from the CD4cre/TNFR2^fl/fl^ and LysMcre/TNFR2^fl/fl^ mouse lines indicating a TNFR2-independent inflammatory reaction upon BCG immunization (data not shown).

In contrast to injections of the TNFR2 agonist into naïve mice, already a single or two injections of TNCscTNF80 given before the last BCG challenge significantly enhanced the frequency of Treg cells in the splenic CD4 T cell population of BCG-immunized mice (Figure 6C). In addition, the activation of TNFR2 enhanced the suppressive activity of Treg cells for CD4 T cell proliferation (Figure 6D). The immune regulatory cytokine IL-10 was also markedly increased in the suppression cultures containing Treg cells from TNCscTNF80 pretreated animals (Figure 6E). However, the frequency of Treg cells in BCG-immunized mice was not altered by two injections of the agonist when given 1 or 3 days after the BCG challenge (data not shown).

## Discussion

Intrigued by recent findings of TNFR2-mediated Treg cell expansion *in vivo* (13, 36), we analyzed the consequences of TNFR2 activation on the cellular level. We focused on three major cell populations: effector T cells, regulatory T cells and myeloid-derived suppressor cells (MDSC).

As we have shown previously, cultures containing GM-CSF and bone marrow cells from TNFR2-deficient mice showed retarded differentiation and a lower yield of mature myeloid cells and reduced nitrite production and suppressive activity of MDSC (21). Unexpectedly, in this study, addition of TNFR2 agonist to cultures of wild-type bone marrow precursor cells had similar effects as TNFR2-deficiency on MDSC. Since endogenous TNF as well as soluble TNFR2, an inhibitor of TNF, are produced in such cultures, the addition of TNCscTNF80 could act as a sink for the soluble TNFR2 thereby enhancing the effect of the endogenous TNF on TNFR1. Alternatively, a possibly bell-shaped response curve of the costimulatory effect could explain these seemingly contradictory results. TNFR2 signaling, however, is unquestionably modulating generation and suppressive functions of MDSC as well as of Treg cells.

The TNFR2-specific agonist TNCscTNF80 improved activation of CD4 and CD8 T cells confirming earlier findings (31, 32). CD4 T cells and, even more sensitive, CD8 T cells showed stronger proliferation upon agonistic TNFR2 activation. TNFR2 expression was required for the TNFR2 agonistic signaling and, as expected, human TNF was not able to induce this costimulatory effect (31). TNCscTNF80, therefore, is a veritable TNFR2-specific agonist providing a costimulatory signal to the T cell receptor. In our hands, this TNFR2-specific costimulation was not able to compensate for the lack of CD28 activation by CD80/CD86. By contrast, Kim and Teh (32) previously suggested that TNFR2 might provide costimulation for CD28-independent T cell activation. However, that study employed a markedly different methodology, such as no specific blockade of CD28 or CD80/CD86, much higher concentrations of plate-bound anti-CD3 for T cell stimulation and the use of TNFR2-deficient cells instead of TNFR2 activation. Therefore, a direct comparison to our approach is difficult.

In contrast to its costimulatory activity, the TNFR2 agonist TNCscTNF80 reduced suppressive activity of Treg cells *in vitro* while, interestingly, at the same time increased the expression of their CD25 and Foxp3. However, prolonged exposure of Treg to the TNFR2 agonist did not change the expression of CD73, OX40L, and CTLA-4 while downregulating markers known to be involved in Treg functions such as CD25, CD39, and GITR. This might explain the reduced suppressive activity of Treg cells after prolonged exposure to TNCscTNF80. Highly purified Treg cells expanded stronger *in vitro* upon addition of TNFR2 agonist, supporting recent data demonstrating the improved expansion of mouse and human Treg cells *in vitro* by additional activation of TNFR2 (15, 16). The expanded Treg cell population was homogenous and did not change the expression levels of CD25, Foxp3, GITR, and notably also not of TNFR2. However, these results obtained under cell culture conditions might not be predictive for the impact of TNCscTNF80 on the Treg cell population *in vivo*.

The analysis of systemic TNFR2-deficient mice had previously demonstrated that TNFR2 is critical for frequency and function of Treg cells [own data and Ref. (12)]. In this context, TNFR2-bearing myeloid cells might interact with newly generated natural Treg cells in the thymus to influence the generation and level of suppressive activity as suggested previously (14). To find out whether TNFR2-bearing myeloid cells cooperate with TNFR2-bearing T cells for generation of Treg cells with maximal suppressive activity, mice with cell type-specific expression of TNFR2 were used in this study. Such a cooperation has been suggested by the data recently shown by Nguyen and Ehrenstein (35). In this study, the suppressive activity of Treg cells from mice with TNFR2-deficient T cells or with myeloid cells expressing low levels of TNFR2 could not be determined unambiguously in suppression tests *in vitro*. However, TNFR2-bearing T cells were crucial for the induction of T cell suppression *in vivo* while a reduction of the presence of TNFR2 on the surface of myeloid cells had no measurable influence. Thus, TNFR2-activity on myeloid cells does not seem to be necessary for the induction of natural Treg cells.

TNFR2-specific activation by the agonist had distinct effects in naive mice and mice undergoing chronic inflammation (BCG model). First signs of increased myelopoiesis in naïve mice were only observed after repeated treatment with TNCscTNF80 indicating a very mild peripheral inflammatory reaction to specific TNFR2 activation in naïve mice. The agonist showed stronger effects in mice with chronic inflammation, induced by BCG immunization, where already a few applications led to increased Treg cell numbers and function. The characteristically strong increase in myeloid cells in the BCG model, as in other models of chronic inflammation, might mask the effects of TNCscTNF80 on myelopoiesis that was seen in mice in steady state. Thus, chronic inflammation seems to increase the sensitivity of Treg cells for TNFR2 costimulation. Possibly, endogenous TNF produced in inflammation sets the stage for effective Treg cell induction by TNFR2 activation. Several studies point to the impact of inflammation and TNF on the Treg population. Inflammation-induced Treg increase has also been shown in a TNF-induced model of rheumatic arthritis (37). Furthermore, activated effector CD4^+^ T cells can boost Treg cell expansion and suppressive function through TNF as shown by Baeyens et al. (38), and in models of autoimmune diabetes (39) and experimental graft-versus-host disease (40). For human Treg cells, Zaragoza et al. (41) recently suggested that TNF does not impair their function *in vitro*. This finding was challenged by Nie et al. (42), who reinforced the earlier concept that TNF reduces human Treg function. The authors discussed that the findings by Zaragoza et al. might be caused by different methodology, such as Treg purification using a positive selection method that might have led to activation of the Treg. This discussion suggests that the impact of TNF on Treg cell function depends on the current activation state of the Treg cells.

Taken together, our data support the use of a TNFR2-specific agonist to utilize TNFR2-specific functions, such as the previous finding of TNFR2-dependent induction of Treg cell expansion *in vivo* (13). However, the joined analysis of effector T cells, Treg cells, and MDSC in this study highlights the pluripotent function of TNFR2 in costimulation and regulation of multiple immune cell populations. Some effects are seemingly contradictory, such as increasing expansion of Treg cells, but inhibiting their suppressive function *in vitro* at the same time. Hence, the anti-inflammatory *in vivo* effects of a TNFR2 agonist depend on the specific model studied, on the level of inflammation and, therefore, the targeted pathology. For therapeutic TNFR2-agonistic treatment, the balance of costimulating effector T cells and expanding Treg cells requires careful *in vivo* investigation as well as analysis of the disease-specific inflammatory state of the immune system.

## Ethics Statement

Mice were bred and housed in an animal facility with barrier conditions at the University of Regensburg. This study was carried out in accordance with institutional guidelines. The protocol was approved by the district government of Lower Franconia, Würzburg (Az: 54-2532.1-27/10, AZ: 54-2532.1-37/13).

## Author Contributions

Conception and drafting of the article: DM and SM. Performance and analysis of experiments: TS, LF, SW, NM, KM, DZ, DW-S, TH, and HW. Discussions of the data and critical revision of the article: HW, SM, and DM.

## Conflict of Interest Statement

The authors declare that the research was conducted in the absence of any commercial or financial relationships that could be construed as a potential conflict of interest.

## References

[1] Feldmann M Maini RN Lasker Clinical Medical Research Award. TNF defined as a therapeutic target for rheumatoid arthritis and other autoimmune diseases. Nat Med (2003) 9:1245–50. 10.1038/nm939 14520364

[2] Monaco C Nanchahal J Taylor P Feldmann M. Anti-TNF therapy: past, present and future. Int Immunol (2015) 27:55–62. 10.1093/intimm/dxu102 25411043 PMC4279876

[3] Williams RO. Collagen-induced arthritis in mice: a major role for tumor necrosis factor-alpha. Methods Mol Biol (2007) 361:265–84. 10.1385/1-59745-208-4:265 17172717

[4] Robinson WH Genovese MC Moreland LW Demyelinating and neurologic events reported in association with tumor necrosis factor alpha antagonism: by what mechanisms could tumor necrosis factor alpha antagonists improve rheumatoid arthritis but exacerbate multiple sclerosis? Arthritis Rheum (2001) 44:1977–83. 10.1002/1529-0131(200109)44:9<1977::AID-ART345>3.0.CO;2-6 11592357

[5] Ramos-Casals M Brito-Zeron P Soto MJ Cuadrado MJ Khamashta MA. Autoimmune diseases induced by TNF-targeted therapies. Best Pract Res Clin Rheumatol (2008) 22:847–61. 10.1016/j.berh.2008.09.008 19028367

[6] Ko JM Gottlieb AB Kerbleski JF. Induction and exacerbation of psoriasis with TNF-blockade therapy: a review and analysis of 127 cases. J Dermatolog Treat (2009) 20:100–8. 10.1080/09546630802441234 18923992

[7] Apostolaki M Armaka M Victoratos P Kollias G. Cellular mechanisms of TNF function in models of inflammation and autoimmunity. Curr Dir Autoimmun (2010) 11:1–26. 10.1159/000289195 20173385

[8] Van Hauwermeiren F Vandenbroucke RE Libert C. Treatment of TNF mediated diseases by selective inhibition of soluble TNF or TNFR1. Cytokine Growth Factor Rev (2011) 22:311–9. 10.1016/j.cytogfr.2011.09.004 21962830

[9] Cope AP Liblau RS Yang XD Congia M Laudanna C Schreiber RD Chronic tumor necrosis factor alters T cell responses by attenuating T cell receptor signaling. J Exp Med (1997) 185:1573–84. 10.1084/jem.185.9.1573 9151895 PMC2196294

[10] Chen X Bäumel M Männel DN Howard OM Oppenheim JJ. Interaction of TNF with TNF receptor type 2 promotes expansion and function of mouse CD4+CD25+ T regulatory cells. J Immunol (2007) 179:154–61. 10.4049/jimmunol.179.1.154 17579033

[11] Chopra M Riedel SS Biehl M Krieger S von Krosigk V Bauerlein CA Tumor necrosis factor receptor 2-dependent homeostasis of regulatory T cells as a player in TNF-induced experimental metastasis. Carcinogenesis (2013) 34:1296–303. 10.1093/carcin/bgt038 23385062

[12] Chen X Wu X Zhou Q Howard OM Netea MG Oppenheim JJ. TNFR2 is critical for the stabilization of the CD4+Foxp3+ regulatory T cell phenotype in the inflammatory environment. J Immunol (2013) 190:1076–84. 10.4049/jimmunol.1202659 23277487 PMC3552130

[13] Chopra M Biehl M Steinfatt T Brandl A Kums J Amich J Exogenous TNFR2 activation protects from acute GvHD via host T reg cell expansion. J Exp Med (2016) 213:1881–900. 10.1084/jem.20151563 27526711 PMC4995078

[14] Mahmud SA Manlove LS Schmitz HM Xing Y Wang Y Owen DL Costimulation via the tumor-necrosis factor receptor superfamily couples TCR signal strength to the thymic differentiation of regulatory T cells. Nat Immunol (2014) 15:473–81. 10.1038/ni.2849 24633226 PMC4000541

[15] Okubo Y Mera T Wang L Faustman DL. Homogeneous expansion of human T-regulatory cells via tumor necrosis factor receptor 2. Sci Rep (2013) 3:3153. 10.1038/srep03153 24193319 PMC3818650

[16] He X Landman S Bauland SC van den Dolder J Koenen HJ Joosten I. A TNFR2-agonist facilitates high purity expansion of human low purity Treg cells. PLoS One (2016) 11:e0156311. 10.1371/journal.pone.0156311 27224512 PMC4880213

[17] Faustman D Davis M. TNF receptor 2 pathway: drug target for autoimmune diseases. Nat Rev Drug Discov (2010) 9:482–93. 10.1038/nrd3030 20489699

[18] Faustman DL Davis M. TNF receptor 2 and disease: autoimmunity and regenerative medicine. Front Immunol (2013) 4:478. 10.3389/fimmu.2013.00478 24391650 PMC3870411

[19] Zhao X Rong L Zhao X Li X Liu X Deng J TNF signaling drives myeloid-derived suppressor cell accumulation. J Clin Invest (2012) 122:4094–104. 10.1172/JCI64115 23064360 PMC3484453

[20] Sade-Feldman M Kanterman J Ish-Shalom E Elnekave M Horwitz E Baniyash M Tumor necrosis factor-alpha blocks differentiation and enhances suppressive activity of immature myeloid cells during chronic inflammation. Immunity (2013) 38:541–54. 10.1016/j.immuni.2013.02.007 23477736

[21] Polz J Remke A Weber S Schmidt D Weber-Steffens D Pietryga-Krieger A Myeloid suppressor cells require membrane TNFR2 expression for suppressive activity. Immun Inflamm Dis (2014) 2:121–30. 10.1002/iid3.19 25400932 PMC4217546

[22] Zhang L Zhang Z Zhang H Wu M Wang Y. Myeloid-derived suppressor cells protect mouse models from autoimmune arthritis via controlling inflammatory response. Inflammation (2014) 37:670–7. 10.1007/s10753-013-9783-z 24264477

[23] Erickson SL de Sauvage FJ Kikly K Carver-Moore K Pitts-Meek S Gillett N Decreased sensitivity to tumour-necrosis factor but normal T-cell development in TNF receptor-2-deficient mice. Nature (1994) 372:560–3. 10.1038/372560a0 7990930

[24] Shen FW Saga Y Litman G Freeman G Tung JS Cantor H Cloning of Ly-5 cDNA. Proc Natl Acad Sci U S A (1985) 82:7360–3. 10.1073/pnas.82.21.7360 3864163 PMC391344

[25] Rodriguez CI Buchholz F Galloway J Sequerra R Kasper J Ayala R High-efficiency deleter mice show that FLPe is an alternative to Cre-loxP. Nat Genet (2000) 25:139–40. 10.1038/75973 10835623

[26] Lee PP Fitzpatrick DR Beard C Jessup HK Lehar S Makar KW A critical role for Dnmt1 and DNA methylation in T cell development, function, and survival. Immunity (2001) 15:763–74. 10.1016/S1074-7613(01)00227-8 11728338

[27] Clausen BE Burkhardt C Reith W Renkawitz R Förster I. Conditional gene targeting in macrophages and granulocytes using LysMcre mice. Transgenic Res (1999) 8:265–77. 10.1023/A:1008942828960 10621974

[28] Mates L Chuah MK Belay E Jerchow B Manoj N Acosta-Sanchez A Molecular evolution of a novel hyperactive sleeping beauty transposase enables robust stable gene transfer in vertebrates. Nat Genet (2009) 41:753–61. 10.1038/ng.343 19412179

[29] Lutz MB Kukutsch N Ogilvie AL Rossner S Koch F Romani N An advanced culture method for generating large quantities of highly pure dendritic cells from mouse bone marrow. J Immunol Methods (1999) 223:77–92. 10.1016/S0022-1759(98)00204-X 10037236

[30] Chen X Subleski JJ Kopf H Howard OM Männel DN Oppenheim JJ Cutting edge: expression of TNFR2 defines a maximally suppressive subset of mouse CD4+CD25+FoxP3+ T regulatory cells: applicability to tumor-infiltrating T regulatory cells. J Immunol (2008) 180:6467–71. 10.4049/jimmunol.180.10.6467 18453563 PMC2699949

[31] Grell M Becke FM Wajant H Männel DN Scheurich P. TNF receptor type 2 mediates thymocyte proliferation independently of TNF receptor type 1. Eur J Immunol (1998) 28:257–63. 10.1002/(SICI)1521-4141(199801)28:01<257::AID-IMMU257>3.0.CO;2-G 9485205

[32] Kim EY Teh HS. TNF type 2 receptor (p75) lowers the threshold of T cell activation. J Immunol (2001) 167:6812–20. 10.4049/jimmunol.167.12.6812 11739497

[33] Lewis M Tartaglia LA Lee A Bennett GL Rice GC Wong GH Cloning and expression of cDNAs for two distinct murine tumor necrosis factor receptors demonstrate one receptor is species specific. Proc Natl Acad Sci U S A (1991) 88:2830–4. 10.1073/pnas.88.7.2830 1849278 PMC51333

[34] Kim EY Teh HS. Critical role of TNF receptor type-2 (p75) as a costimulator for IL-2 induction and T cell survival: a functional link to CD28. J Immunol (2004) 173:4500–9. 10.4049/jimmunol.173.7.4500 15383581

[35] Nguyen DX Ehrenstein MR. Anti-TNF drives regulatory T cell expansion by paradoxically promoting membrane TNF-TNF-RII binding in rheumatoid arthritis. J Exp Med (2016) 213:1241–53. 10.1084/jem.20151255 27270893 PMC4925013

[36] Pierini A Strober W Moffett C Baker J Nishikii H Alvarez M TNF-alpha priming enhances CD4+FoxP3+ regulatory T-cell suppressive function in murine GVHD prevention and treatment. Blood (2016) 128:866–71. 10.1182/blood-2016-04-711275 27365424 PMC4982455

[37] Biton J Semerano L Delavalle L Lemeiter D Laborie M Grouard-Vogel M Interplay between TNF and regulatory T cells in a TNF-driven murine model of arthritis. J Immunol (2011) 186:3899–910. 10.4049/jimmunol.1003372 21346237

[38] Baeyens A Saadoun D Billiard F Rouers A Grégoire S Zaragoza B Effector T cells boost regulatory T cell expansion by IL-2, TNF, OX40, and plasmacytoid dendritic cells depending on the immune context. J Immunol (2015) 194:999–1010. 10.4049/jimmunol.1400504 25548233

[39] Grinberg-Bleyer Y Saadoun D Baeyens A Billiard F Goldstein JD Grégoire S Pathogenic T cells have a paradoxical protective effect in murine autoimmune diabetes by boosting Tregs. J Clin Invest (2010) 120:4558–68. 10.1172/JCI42945 21099113 PMC2993590

[40] Leclerc M Naserian S Pilon C Thiolat A Martin GH Pouchy C Control of GVHD by regulatory T cells depends on TNF produced by T cells and TNFR2 expressed by regulatory T cells. Blood (2016) 128:1651–9. 10.1182/blood-2016-02-700849 27506541

[41] Zaragoza B Chen X Oppenheim JJ Baeyens A Gregoire S Chader D Suppressive activity of human regulatory T cells is maintained in the presence of TNF. Nat Med (2016) 22:16–7. 10.1038/nm.4019 26735402 PMC6345394

[42] Nie H Zheng Y Li R Zhang J Reply to suppressive activity of human regulatory T cells is maintained in the presence of TNF. Nat Med (2016) 22:18–9. 10.1038/nm.4018 26735403

